# 既往吉非替尼治疗获益的晚期NSCLC患者再次使用EGFR-TKI的选择：原药还是换药？

**DOI:** 10.3779/j.issn.1009-3419.2013.07.03

**Published:** 2013-07-20

**Authors:** 传昊 汤, 晓燕 李, 万峰 郭, 俭杰 李, 海峰 秦, 伟霞 王, 莉莉 曲, 娟 安, 红军 高, 晓晴 刘

**Affiliations:** 100071 北京，军事医学科学院附属医院肺部肿瘤科 Department of Lung Cancer, Affiliated Hospital of Academy of Military Medical Sciences, Beijing 100071, China

**Keywords:** 肺肿瘤, 表皮生长因子受体, 吉非替尼, 厄洛替尼, Lung neoplasms, Epidermal growth factor receptor, Gefitinib, Erlotinib

## Abstract

**背景与目的:**

既往表皮生长因子受体酪氨酸激酶抑制剂（epidermal growth factor receptor tyrosine kinase inhibitor, EGFR-TKI）治疗获益的晚期非小细胞肺癌（non-small cell lung cancer, NSCLC）患者，再次给予TKI治疗，已逐渐成为一种新的治疗策略。本研究旨在探讨二次TKI治疗时，原药或换药，哪一种选择更为合理。

**方法:**

回顾晚期或术后复发的NSCLC患者，既往吉非替尼治疗疗效达到完全缓解（complete response, CR）、部分缓解（partial response, PR）或稳定（stable disease, SD），无进展生存期（progression free survival, PFS）≥3个月，病情进展后，间隔时间至少1个月，分别接受吉非替尼或厄洛替尼治疗。就两组患者的疗效、优势人群等进行分析。

**结果:**

共有61例患者入组，其中吉非替尼组30例，厄洛替尼组31例，两组患者基线特征基本平衡。吉非替尼组与厄洛替尼组疗效比较，有效率（response rate, RR）（10% *vs* 22.6%, *P*=0.300, 6）、疾病控制率（disease contral rate, DCR）（60% *vs* 74.2%, *P*=0.237, 8）、中位PFS（3.0个月*vs* 3.5个月，*P*=0.494, 5）、中位总生存期（overall survival, OS）（8.3个月*vs* 8.5个月，*P*=0.140, 8）均未见统计学差异。多因素分析示：首次吉非替尼PFS≥6个月（HR=0.317, 95%CI: 0.102-0.984, *P*=0.046, 9），两次TKI间隔时间≥3个月（HR=0.224, 95%CI: 0.071-0.713, *P*=0.011, 3）的患者疾病进展风险降低。而两次TKI间隔时间≥3个月（HR=0.262, 95%CI: 0.097-0.705, *P*=0.008, 0）的患者死亡风险降低。

**结论:**

既往吉非替尼治疗获益的晚期NSCLC患者再次TKI治疗，无论选择吉非替尼还是换用厄洛替尼均可获益，这种获益与首次TKI的PFS、以及两次TKI的间隔时间相关。

## Abstract (en)

**Background and objective:**

For advanced non-small cell lung cancer (NSCLC) patients who benefited from prior epidermal growth factor receptor tyrosine kinase inhibitor (EGFR-TKI) therapy, the choice of a second TKI therapy has gradually become a new strategy for the treatment. Some investigators recommend that the second therapy should be continued with the original TKI; however, other investigators recommend the administration of another TKI. The aim of this study is to explore which choice is more reasonable.

**Methods:**

In retrospect, patients with advanced NSCLC or with postoperative relapse of advanced NSCLC achieved complete response (CR), partial response (PR) or stable disease (SD) in prior Gefitinib therapy, progression free survival (PFS) ≥3 months. They received repeated Gefitinib or Erlotinib at an interval of at least one month. The analysis was carried out with respect to efficacy and optimal population of the two groups.

**Results:**

A total of 61 patients were enrolled into the study, 30 in Gefitinib group and 31 in Erlotinib group. Baseline characteristics of the two groups were comparable. In the comparison between patients treated with Gefitinib and with Erlotinib, no statistical differences were seen for response rate (10% *vs* 22.6%, *P*=0.300, 6), disease control rate (60% *vs* 74.2%, *P*=0.237, 8), median PFS (3.0 *vs* 3.5 months, *P*=0.494, 5), or median OS (8.3 *vs* 8.5 months, *P*=0.140, 8). Multivariate analysis showed that in the initial dose of Gefitinib, PFS≥6 months (HR=0.317, 95%CI: 0.102-0.984, *P*=0.046, 9). With an interval ≥3 months (HR=0.224, 95%CI: 0.071-0.713, *P*=0.011, 3) between two doses of TKI, the risk of disease progression was reduced; but if with an interval ≥3 months (HR=0.262, 95%CI: 0.097-0.705, *P*=0.008, 0), the risk of death was reduced.

**Conclusion:**

Advanced NSCLC patients who benefited from prior Gefitinib therapy can benefit again either with the original drug Gefitinib or the alternative drug Erlotinib when a second TKI therapy is resumed. Such benefit is related to PFS of initial TKI therapy and time interval between two doses of TKI.

以吉非替尼和厄洛替尼为代表的表皮生长因子受体酪氨酸激酶抑制剂（epidermal growth factor receptor tyrosine kinase inhibitor, EGFR-TKI）在晚期非小细胞肺癌（non-small cell lung cancer, NSCLC）个体化治疗中应用广泛。各种指南推荐的临床适应症涵盖了晚期NSCLC化疗失败后的标准二、三线治疗^[1, 2]^、*EGFR*基因敏感突变的一线治疗^[3, 4]^以及维持治疗^[5, 6]^。对于优势人群，使用EGFR-TKI不但可以达到长期疾病无进展生存，总生存期也由传统化疗时代1年左右的时间提高到2年以上^[3, 4]^。来自NEJ002研究的更新数据^[7]^显示，*EGFR*基因敏感突变的晚期NSCLC患者给予吉非替尼及含铂两药方案化疗，中位生存期接近3年。对于这些长期生存的患者，由于前期已然接受过包括EGFR-TKI和铂二联化疗在内的多线治疗，当病情再次进展时，NCCN指南已无推荐。除了入组新药临床研究外，临床医生尝试对那些既往EGFR-TKI获益的患者，二次给予TKI治疗，证实仍有部分患者可再次从中获益^[8-13]^。一项来自日本的回顾性研究^[13]^显示接受过两次TKI治疗的患者总生存期获得明显延长。或许二次TKI治疗在今后临床中的应用将越来越广泛。对于二次TKI的用药选择目前尚有争议，部分研究者建议继续给予原TKI治疗^[8, 11-13]^，但也有研究者认为应换用另外一种TKI^[9, 10]^。本文是一篇回顾性研究，旨在探讨既往吉非替尼治疗获益的患者，二次选择TKI治疗时究竟是继续给予吉非替尼治疗，还是换用厄洛替尼治疗，哪种选择更为合理。

## 1 材料与方法

### 1.1 病例收集

回顾2005年4月-2012年8月期间，在解放军第三〇七医院肺部肿瘤科就诊，接受过两次TKI治疗的晚期或术后转移复发的NSCLC患者。入选标准：①首次TKI为吉非替尼; ②首次TKI疗效为完全缓解（complete response, CR）、部分缓解（partial response, PR）或稳定（stable disease, SD）; ③首次TKI的PFS≥3个月; ④首次TKI治疗失败以后再次接受吉非替尼或厄洛替尼治疗; ⑤两次TKI间隔时间≥1个月。

### 1.2 治疗和评价方法

吉非替尼的用法为250 mg/d，厄洛替尼用法为150 mg/d，28天为1周期，用药1周期行首次疗效评价，此后每2周期复查评估。

疗效评价依据RECIST（Response Evaluation Criteria in Solid Tumors）1.0标准，无进展生存期（progression free survival, PFS）定义为自接受EGFR-TKI治疗开始，至观察到疾病进展或者发生因为任何原因死亡的时间。中位总生存期（overall survival, OS）定义为自接受EGFR-TKI治疗开始至发生因为任何原因死亡的时间。二次TKI毒副反应判定参考NCI-CTC（National Cancer Institute Common Toxicity Criteria）3.0标准。

### 1.2 统计方法

SAS 9.2软件进行统计分析。采用卡方检验或*Fisher*精确概率法比较有效率和疾病控制率。*Kaplan-Meier*法描绘生存曲线，*Log-rank*法检验不同组间差异。多因素分析采用*Cox*比例风险回归模型评价不同基线特征与进展及生存预后关系。*P* < 0.05为差异有统计学意义。

## 2 结果

### 2.1 患者特征

共有61例符合入组条件的患者纳入本研究。中位年龄57岁（33岁-77岁），病理类型均为腺癌，临床分期均为Ⅳ期。按照二次TKI药物选择分组，吉非替尼组30例，厄洛替尼组31例。两组患者年龄、性别、吸烟状况、ECOG评分、*EGFR*基因检测情况、远处转移器官的数目及部位，首次吉非替尼的治疗时机、首次吉非替尼的缓解情况及PFS、两次TKI的间隔时间及间隔治疗等基线特征基本平衡（表 1）。

**1:** 入组患者基线特征 Baseline characteristics of included patients

Characteristics	Gefitinib group (*n*=30)	Erlotinib group (*n*=31)	*P*
Age (yr)			
Median (range)	55 (33-74)	57 (33-77)	0.519, 3
≥65	10 (33.33%)	8 (25.81%)	
< 65	20 (66.67%)	23 (74.19%)	
Gender			0.715, 0
Male	9 (30.00%)	8 (25.81%)	
Female	21 (70.00%)	23 (74.19%)	
Smoking status			0.507, 7
Never smoker	24 (80.00%)	27 (87.10%)	
Current or ever smoker	6 (20.00%)	4 (12.90%)	
ECOG			0.899, 8
0-1	15 (50.00%)	15 (48.39%)	
≥2	15 (50.00%)	16 (51.61%)	
*EGFR* mutation			0.321, 5
19(+)	19 (63.33%)	13 (41.94%)	
21(+)	1 (3.33%)	1 (3.23%)	
Wild	1 (3.33%)	3 (9.68%)	
Not available	9 (30.00%)	14 (45.16%)	
Number of metastasized organs			0.859, 7
1-2	20 (66.67%)	20 (64.52%)	
≥3	10 (33.33%)	11 (35.48%)	
Metastasized region			
Lung	22 (73.33%)	25 (80.65%)	0.497, 2
Brain	15 (50.00%)	15 (48.39%)	0.899, 8
Bone	12 (40.00%)	15 (48.39%)	0.509, 7
Liver	5 (16.67%)	7 (22.58%)	0.211, 9
Initial TKI			0.123, 5
1^st^ line	11 (36.67%)	8 (25.81%)	
2^nd^ line	17 (56.67%)	15 (48.39%)	
≥3^rd^ line	2 (6.67%)	8 (25.81%)	
Response of initial TKI			0.437, 0
CR+PR	23 (76.67%)	21 (67.74%)	
SD	7 (23.33%)	10 (32.26%)	
PFS of initial TKI			0.195, 3
≥6 months	29 (96.67%)	26 (83.74%)	
< 6 months	1 (3.33%)	5 (16.13%)	
Interval chemotherapy			0.347, 6
Yes	26 (86.67%)	24 (77.42%)	
No	4 (13.33%)	7 (22.58%)	
Interval time			0.785, 0
≥3 months	25 (83.33%)	25 (80.65%)	
< 3 months	5 (16.67%)	6 (19.35%)	
ECOG: Eastern Cooperative Oncology Group; PS: performance status; EGFR: epidermal growth factor receptor; TKI: tyrosine kinase inhibitor.			

### 2.2 二次使用EGFR-TKI疗效

吉非替尼组与厄洛替尼组RR和DCR分别为10% *vs* 22.6%（*P*=0.300, 6）和60% *vs* 74.2%（*P*=0.237, 8）（表 2）。中位OS吉非替尼组8.3个月*vs*厄洛替尼组8.5个月（*P*=0.140, 8）（图 1）。中位PFS吉非替尼组3.0个月*vs*厄洛替尼组3.5个月（*P*=0.494, 5）（图 2）。

**2:** 吉非替尼组和厄洛替尼组疗效 Tumor response in gefitinib-treated and erlotinib-treated patients

Response	Gefitinib group (*n*=30)	Erlotinib group (*n*=31)	*P*
CR	0	0	
PR	3	7	
SD	15	16	
PD	12	8	
DCR	60.00%	74.19%	0.237, 8
RR	10.00%	22.58%	0.300, 6
CR: complete response; PR: partial response; SD: stable disease; PD: progressive disease; DCR: disease control rate; RR: response rate.			

**1:**
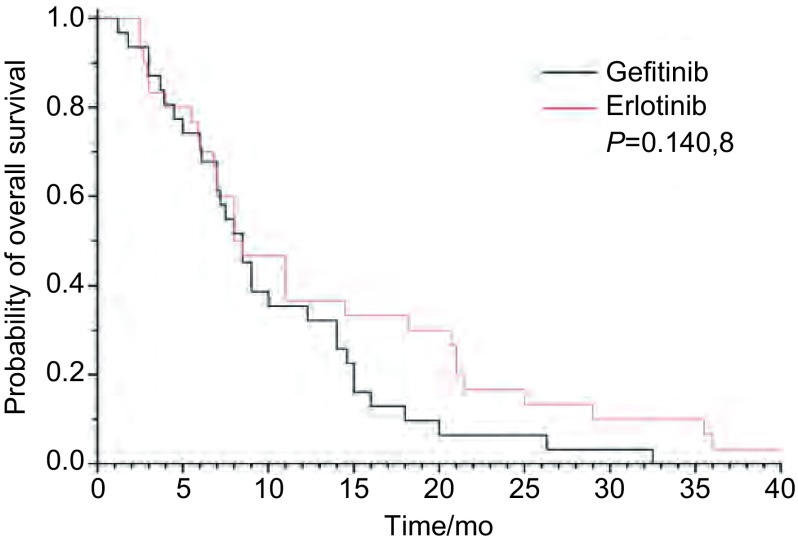
吉非替尼组与厄洛替尼组总生存曲线 Comparison of *Kaplan-Meier* curves is shown for overall survival (OS) between patients treated with gefitinib and with erlotinib

**2:**
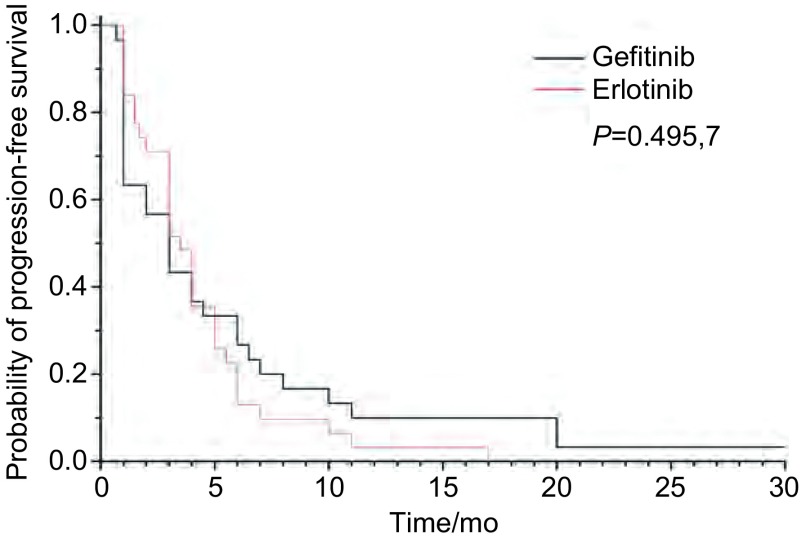
吉非替尼组与厄洛替尼组无进展生存曲线 Comparison of *Kaplan-Meier* curves is shown for progression free survival (PFS) between patients treated with gefitinib and with erlotinib

入组的61例患者RR为16.4%（10/61），DCR为67.2%（41/61），中位OS为8.5个月，1年生存率为36.1%，中位PFS为3.5个月。

OS相关的多因素分析提示两次TKI间隔时间≥3个月（HR=0.262, 95%CI: 0.097-0.705, *P*=0.008, 0）是降低死亡风险的独立预后因子（表 3）。

**3:** OS多因素分析 Multivariate analysis of overall survival

Variable	Patients *n* (%)	Median OS (mo)	*P*	HR (95%CI)
Age (yr)			0.217, 6	0.641 (0.316-1.300)
≥65	18	7.6		
< 65	43	9.0		
Gender			0.971, 8	0.982 (0.360-2.677)
Male	17	8.0		
Female	44	8.5		
Smoking status			0.840, 7	0.880 (0.252-3.074)
Never	51	8.5		
Current or ever	10	8.0		
ECOG			0.177, 7	0.637 (0.379-1.197)
0-1	30	11.0		
≥2	31	7.5		
*EGFR* mutation			0.321, 7	0.653 (0.346-1.289)
(+)	34	8.0		
(-)	4	9.8		
Number of metastasized organs			0.425, 2	0.706 (0.300-1.611)
1-2	40	8.5		
≥3	21	8.0		
Metastasized region				
Lung	47	9.0	0.471, 5	0.750 (0.343-1.640)
Brain	30	7.25	0.492, 5	0.777 (0.377-1.599)
liver	12	8.75	0.245, 8	0.608 (0.262-1.408)
Bone	27	7.2	0.517, 1	0.801 (0.409-1.568)
Response of initial TKI			0.490, 1	0.755 (0.339-1.681)
CR+PR	44	8.5		
SD	17	8.0		
PFS of initial TKI			0.217, 3	0.456 (1.131-1.588)
≥6 months	55	8.5		
< 6 months	6	12.15		
Interval chemotherapy			0.866, 9	0.971 (0.331-2.535)
Yes	50	9.0		
No	11	5.0		
Interval time			0.008, 0	0.262 (0.097-0.705)
≥3 months	50	9.0		
< 3 months	11	5.0		
OS: overall survival; CI: confidence interval; HR: hazard ratio.				

PFS相关的多因素分析提示首次吉非替尼PFS≥6个月（HR=0.317, 95%CI: 0.102-0.984, *P*=0.046, 9），两次TKI间隔时间≥3个月（HR=0.224, 95%CI: 0.071-0.713, *P*=0.011, 3）是降低疾病进展风险的独立预后因子（表 4）。

**4:** PFS多因素分析 Multivariate analysis of progression-free survival

Variable	Patients *n* (%)	Median PFS (mo)	*P*	HR (95%CI)
Age (yr)			0.314, 0	0.701 (0.350-1.400)
≥65	18	3.0		
< 65	43	3.5		
Gender			0.937, 0	0.960 (0.353-2.610)
Male	17	3.0		
Female	44	3.0		
Smoking status			0.276, 0	0.531 (0.170-1.657)
Never	51	3.0		
Current or ever	10	2.5		
ECOG			0.433, 8	0.797 (0.451-1.407)
0-1	30	4.0		
≥2	31	3.0		
*EGFR* mutation			0.148, 3	0.677 (0.324-1.211)
(+)	34	3.5		
(-)	4	2.35		
Number of metastasized organs			0.766, 6	0.871 (0.350-2.169)
1-2	40	4.0		
≥3	21	3.0		
Metastasized region				
Lung	47	4.0	0.719, 0	0.719 (0.371-1.394)
Brain	30	3.0	0.052, 0	0.477 (0.266-1.007)
Liver	12	3.0	0.576, 4	0.797 (0.360-1.766)
Bone	27	3.0	0.117, 6	0.553 (0.264-1.161)
Response of initial TKI			0.977, 7	0.990 (0.482-2.034)
CR+PR	44	3.75		
SD	17	2.0		
PFS of initial TKI			0.046, 9	0.317 (0.102-0.984)
≥6 months	55	4.0		
< 6 months	6	1.0		
Interval chemotherapy			0.112, 7	0.398 (0.127-1.243)
Yes	50	4.0		
No	11	1.5		
Interval time			0.011, 3	0.224 (0.071-0.713)
≥3 months	50	4.0		
< 3 months	11	1.0		

### 2.3 不良反应

厄洛替尼组皮疹的发生率要高于吉非替尼组（64.51% *vs* 36.67%, *P*=0.030, 1），其余发生率超过10%的不良反应，两组均未见统计学差异（表 5）。全组发生Ⅲ度-Ⅳ度不良反应仅3例，其中皮疹2例，咯血1例，均发生在厄洛替尼组，给予厄洛替尼减量及对症治疗后症状缓解。

**5:** 吉非替尼组和厄洛替尼组不良反应 Adverse effects in gefitinib-treated and erlotinib-treated patients

Adverse effect	Gefitinib group (*n*=30)	Erlotinib group (*n*=31)	*P*
Rash	11 (36.67%)	20 (64.51%)	0.030, 1
Diarrhea	7 (23.33%)	7 (22.58%)	0.944, 3
Asthenia	5 (16.67%)	4 (12.90%)	0.679, 2
Anorexia	5 (16.67%)	5 (16.13%)	0.955, 3
Dry skin	4 (13.33%)	4 (12.90%)	0.969, 0
Nausea/vomiting	3 (10.00%)	4 (12.90%)	0.722, 4
Hand-foot syndrome	3 (10.00%)	3 (9.67%)	0.966, 0
Stomatitis	3 (10.00%)	2 (6.45%)	0.785, 2

## 3 讨论

二次给予EGFR-TKI治疗已经为越来越多的临床医生关注和实践，近年来亦有多篇个案和小样本回顾性研究报道。对于二次TKI药物的选择问题，目前还存有一定的争议，究竟是继续选择原TKI治疗还是换用另外一种TKI，尚缺乏循证医学证据。

继续选择原TKI治疗的前提是必须从首次TKI治疗中明显获益，经过1个化疗“间歇期”，有部分患者可重新恢复对EGFR-TKI的敏感性。一项单臂、开放、前瞻性的Ⅱ期临床研究^[12]^，23例Ⅳ期NSCLC患者首次接受吉非替尼治疗有效或稳定的患者，PFS≥3个月，进展后至少接受一种细胞毒药物化疗，再次进展后仍给予吉非替尼治疗，RR为21.7%（5例），DCR为65.2%（13例），中位TTP为103天，中位OS为343天。

一种TKI治疗失败后换用另外一种TKI多见于吉非替尼失败后换用厄洛替尼。同样作为小分子酪氨酸激酶抑制剂，两种药物有相近的分子结构和作用机理。但在常规推荐浓度下，厄洛替尼的血药浓度和生物学活性要高于吉非替尼，并且基础研究^[10]^提示，提高TKI的药物浓度有可能克服继发耐药。Kaira等^[9]^对11组研究共106例吉非替尼失败后厄洛替尼治疗的患者进行汇总分析，厄洛替尼治疗的PR、SD、DCR分别为9.9%、18.9%和28.8%。鉴于汇总的病例中有相当数量的患者既往吉非替尼未获益，或吉非替尼治疗失败后立即更换为厄洛替尼治疗，从而影响了二次TKI的疗效数据，因此作者没有把这种换药模式作为推荐。但依据优势人群分析，既往吉非替尼疗效部分缓解或稳定，PFS≥6个月，吉非替尼失败后停药时间超过3个月接受厄洛替尼治疗获益明显。

在本研究中，我们筛选既往吉非替尼治疗明显获益的晚期NSCLC患者，疗效CR、PR或SD，PFS≥3个月。且均是吉非替尼治疗失败，间隔一段时间后，再接受二次TKI治疗。在患者基线条件基本均衡的情况下，将原药组与换药组各自疗效进行对比分析。结果显示：厄洛替尼组的RR和DCR略高于吉非替尼组，22.6% *vs* 10%和74.2% *vs* 60%，但未达统计学差异。两组的中位PFS和中位OS数据相近，同样未见统计学差异。多因素分析的结果与以往报道一致，首次TKI的PFS≥6个月和两次TKI间隔时间≥3个月是二次TKI治疗获益的优势人群。因此我们认为，二次TKI能否获益关键在于首次TKI的疗效和足够长的停药时间，而可能与选择何种TKI药物无关，当然这一结论尚需前瞻性研究进一步验证。

对于EGFR-TKI获得性耐药的机理目前已有一定共识：约50%为T790M二次突变; 20%为*c-MET*基因扩增，还有约30%的其它已知和不明分子机制^[14, 15]^。而二次TKI获益的机理目前尚不十分明确。有研究者通过重复活检实时检测发现，*EGFR*敏感突变患者，TKI获得性耐药给予化疗等其它专科治疗，一段时间之后，敏感突变可再次出现^[16]^。另有研究者认为，患者再次对TKI治疗产生反应可能与肿瘤异质性相关，初次对靶向药物治疗发生反应后，可能有小部分EGFR-TKI依赖性肿瘤细胞残留，随时间推移，肿瘤可能重新被这些细胞占据而再次出现TKI获益^[17]^。无论是肿瘤细胞通路自身调节的结果还是肿瘤异质性的原因，都为二次选择TKI治疗的可行性提供了理论依据。这其中一个重要的前提就是首次TKI耐药后停药时间需尽量长，多篇文献建议两次TKI间隔时间应≥3个月。本文也再次证实，间隔时间≥3个月不但降低了进展风险而且还是生存获益的独立预后因素。

二次使用EGFR-TKI的不良反应与既往多个Ⅱ期和Ⅲ期EGFR-TKI相关的临床研究报道相似，最常见的依然是皮疹和腹泻，多为Ⅰ度-Ⅱ度。其中厄洛替尼组皮疹的发生率要高于吉非替尼组。

本文是一项回顾性研究，首次就既往吉非替尼治疗获益的患者二次TKI治疗的用药选择进行探讨。结论提示，无论继续选择吉非替尼还是换用厄洛替尼均可获益，首次吉非替尼PFS≥6个月、两次TKI间隔时间≥3个月是独立的预后因子。我们期待更多的大样本前瞻性研究，以期更合理的选择二次TKI治疗的用药，确定优势人群和最佳治疗时机，从而达到获取更长生存期的目的。
